# Procedural and clinical outcomes of Adiana® hysteroscopic tubal occlusion in the Netherlands

**DOI:** 10.52054/FVVO.16.4.050

**Published:** 2024-12-27

**Authors:** D.M. van Gastel, L.W. Maassen, M.A.J.M. Van Erp, A.L.W.M. Coolen, A.L. Thurkow, C.A.M. Koks, S Veersema, M.Y. Bongers

**Affiliations:** University Maastricht, Researchschool Grow, P. Debyelaan 25, 6229 HX Maastricht, The Netherlands; University Medical Center Utrecht, Heidelberglaan 100, 3584 CX Utrecht, The Netherlands; Bergman Clinics Amsterdam, Amsterdam, The Netherlands; Máxima Medical Center Veldhoven, De Run 4600, 5504 DB Veldhoven, The Netherlands

**Keywords:** Adiana, hysteroscopic tubal occlusion, permanent female contraception, outpatient sterilisation

## Abstract

**Background:**

The Adiana® Permanent Contraception System was a hysteroscopic tubal occlusion device but was withdrawn from the market in 2012.

**Objective:**

To evaluate the safety, feasibility and efficacy of the Adiana hysteroscopic tubal occlusion.

**Materials and Methods:**

A prospective observational multicentre cohort study of 300 women undergoing hysteroscopic sterilisation using the Adiana® was conducted in the Netherlands between 2009 and 2012. All procedures were performed using the same study protocol. Three months after bilateral placement a hysterosalpingography (HSG) was performed to confirm tubal occlusion. In 2018-2020 follow-up questionnaires were sent to all women.

**Main outcome measures:**

The primary outcome was the success rate of the Adiana tubal occlusion technique. Successful tubal occlusion was defined as an uneventful procedure with occluded fallopian tubes according to the HSG after 3 months. Secondary outcomes were the success rate of the device placement, the number of complications during placement and the pregnancy rate.

**Results:**

Bilateral placement of Adiana devices was achieved in 93.5% of cases. Bilateral confirmed occlusion by HSG was accomplished in 87.9% of cases with successful Adiana placement. This was 77.1% in the intention- to-treat group. Complications and side effects were reported in 4.4% of women. The pregnancy rate was 3.6% in women with proven bilateral tubal occlusion.

**Conclusion:**

Hysteroscopic tubal occlusion using the Adiana technology is associated with a pregnancy rate of 3.6%. Although this technology was removed from the commercial market, this evaluation of the Adiana technology could provide useful information for the development of potential new, more effective hysteroscopic tubal occlusion devices.

**What’s new?:**

Hysteroscopic tubal occlusion techniques are no longer available on the market. This evaluation of Adiana could provide useful information for the development of potential new hysteroscopic tubal occlusion devices.

## Introduction

Adiana® Permanent Contraception System (Adiana Inc., Redwood City, CA purchased by Hologic, USA) was a hysteroscopic tubal occlusion device, approved by the United States Food and Drug Administration (FDA) in 2009. In March 2012 a long-standing battle over patent infringement between the companies Conceptus (Essure ®) and Hologic (Adiana®) took place. Eventually, Adiana was withdrawn from the market in 2012 to settle the alleged patent infringement litigation (Veersema, 2015).

Adiana tubal occlusion was a two-stage procedure. First, bipolar radiofrequency energy was used to create a lesion of the tubal endothelium whereafter a 3.5-mm silicone, non-biodegradable porous implant was positioned into the tubal lumen.

As a result of a subsequent fibrous reaction, the tubes became occluded. The effectiveness of the Adiana micro insert in preventing pregnancy was believed to be due to a combination of the space- filling design and a local, occlusive, benign tissue response to the silicon implant. A thermal lesion activated the inflammatory and fibrotic response and was meant to fixate the Adiana device in the fallopian tube (Vancaillie et al., 2008; Herbst and Evantash, 2010).

The first evaluation studies – the EASE trial (Evaluation of the Adiana System for Transcervical Sterilization) - showed high satisfaction and comfort scores after Adiana sterilisation (Vancaillie et al., 2008; Anderson and Vancaillie, 2011). The efficacy was described as a cumulative pregnancy prevention rate after three years of 98.4% (Anderson and Vancaillie, 2011). There were no reports of discomfort, acute pain or bleeding associated with the Adiana devices. Reported adverse events occurred in less than 5% of cases, of which dysmenorrhea was most reported. A serious adverse event reported after Adiana tubal occlusion was an ectopic pregnancy (Anderson and Vancaillie, 2011). Besides Adiana, two other hysteroscopic tubal occlusion devices were commercially available in the last decades: Ovabloc® Intra Tubal Device (Advanced Medical Grade Silicones BV) and the Essure ® device (Conceptus Incorporated; later Bayer AG) (La Chapelle et al., 2015). However, all devices turned out to have limitations leading to withdrawal from the market.

Hysteroscopic techniques are beneficial compared to laparoscopic methods: there is no need for general anaesthesia, they can be performed in an outpatient setting, they leave no scars, and the complication risks are low. Further advantages include a rapid recovery, and it is an alternative for women for whom laparoscopy is not favourable or even contra-indicated. In the Netherlands, a prospective multicentre research trial regarding Adiana tubal occlusion was conducted from 2009 to 2012 to investigate the placement procedure, success rate, complication rate and short-term follow-up. Concomitant with the withdrawal of Adiana from the market, this study was discontinued, and results were not published. For women seeking permanent contraception, hysteroscopic tubal occlusion techniques have great benefits over laparoscopic sterilisation methods. The aim of this study was to evaluate the success rate of the Adiana hysteroscopic tubal occlusion technique in an outpatient, daily practice setting. Since all hysteroscopic techniques are no longer available due to limitations and legal issues, we present this data to analyse the advantages and disadvantages of previously used methods, with the aim of drawing lessons that could inform the future development of sterilization devices.

## Methods

A prospective multicentre trial was performed in six teaching hospitals between 2009 and 2012 in the Netherlands. Exclusion criteria were contra-indications for sterilisation, age under 25 or an incomplete family, contra-indications for hysteroscopy, medical history of unilateral tubectomy and the presence of active infection or pregnancy. Included patients were women consulted for sterilisation, who had chosen the Adiana technique after receiving information about laparoscopic sterilisation, the hysteroscopic tubal occlusion techniques Essure and Adiana. The advantages and disadvantages of all options were explained, such as the outpatient setting and, absence of postoperative scars. They were also informed about the low complication rate, time of the procedure and short recovery time. The limited experience with the Adiana procedure was discussed. If the Adiana System was the treatment of choice by the patient, women were scheduled for an Adiana tubal occlusion in the proliferative phase of the menstrual cycle or during the use of oral contraceptives.

At the time of the study, obtaining ethical approval of the Medical Research Involving Human Subjects Act (WMO) was not yet required and therefore not included.

The procedure was performed by nine experienced senior gynaecologists, in six teaching hospitals, who all completed a special Adiana tubal occlusion training. The training was organised by Hologic and experienced hysteroscopists explained the procedure. Training models were used to practice. In all clinics, the gynaecologists followed the same study protocol. The Adiana tubal occlusion was performed hysteroscopically via the vaginoscopic technique without the use of a speculum. A hysteroscopic shaft with a 5 French working channel and a 3mm telescope was used. Women were advised to take Naproxen (500 mg oral) one to two hours prior to the procedure.

The Adiana ® sterilisation method was a combination of a controlled thermal lesion to the lining of the fallopian tube followed by the insertion of a non-absorbable biocompatible silicone elastomer matrix within the tubal lumen. The Adiana® sterilisation technique has three components: a radiofrequency generator, a delivery catheter and an implantable device.

The delivery catheter, designed to be introduced through a 5 French working channel of an operative hysteroscope, was used to access the fallopian tubes and deliver the Adiana® devices into the intramural portion of the fallopian tube. The delivery catheter included a bipolar electrode array and Position Detection Array (PDA) to signal the physician that the catheter was in a tubular structure. The PDA was a series of four sensors that were designed to monitor uniform tissue contact throughout the ablation portion of the procedure. Under hysteroscopic guidance, the delivery catheter was introduced into the tubal ostium. Proper deployment of the Adiana® delivery catheter was identified by a black marker at the tubal ostia and through the feedback provided by the PDA.

Once placement inside the intramural section of the fallopian tube was confirmed, the distal tip of the catheter delivered radiofrequency (RF) energy for a period of 1 minute, causing a 5-mm thermal lesion within the fallopian tube. The RF generator was programmed to reach and maintain a temperature of 64oC at the catheter tip. Following thermal injury, the 3.5-mm silicone matrix was deployed within the lesion and the catheter and hysteroscope were removed. The matrix is a cylindrical piece of porous silicone with a solid core, approximately 3.5 mm long and 1.5 mm in diameter. No portion of the device extends into the uterine cavity that could interfere with any future gynaecologic procedures. Over the next few months, occlusion was achieved by fibroblast ingrowth into the porous matrix which completely occludes the tubal lumen.

According to the official Adiana protocol, all included women were scheduled for hysterosalpingography (HSG) three months after bilateral device placement to confirm bilateral tubal occlusion. Until bilateral occlusion of the fallopian tubes was confirmed, all women were advised to use alternative contraception. If bilateral occlusion was not confirmed after three months, a second HSG was performed after six months. If tubal occlusion was confirmed by HSG, successful tubal occlusion was assumed, and women were instructed to rely on the Adiana tubal occlusion as contraception. Initially, the Adiana device was not radiopaque and therefore not visible on the HSG. Adiana devices were detectable on transvaginal ultrasonography which was performed three months after Adiana tubal occlusion. In line with the official Adiana protocol, no clinical management decisions were made upon ultrasound results. The reason for the ultrasound was an extra check of the devices regarding localisation and for further research as a possible replacement of the HSG.

Successful sterilisation as primary outcome was defined as bilateral occlusion of the fallopian tubes, confirmed by HSG. Secondary outcomes were success rate of device placement, complications during placement and pregnancies. Data collected from each woman included age, gravidity, parity, intra-uterine disorders, placement of one or both devices, the number of procedures needed for bilateral placement, and the performance of another hysteroscopic treatment during the same surgery. In case of failure, the reason was documented.

Since this data was not published earlier, we sent out questionnaires between February 2018 and December 2020 to all women in the Netherlands (N=300) who underwent Adiana® sterilisation. The research ethics committee of the Maxima Medical Centre declared that this study did not fall within the remit of the Medical Research Involving Human Subjects Act (WMO). Ethical approval for this multi-centre retrospective cohort study was obtained from the institutional ethics committees of participating hospitals under number N17.172.

Questionnaires included questions about satisfaction with Adiana® devices, removal of devices, the necessity of hysterectomy and the occurrence of pregnancies.

## Results

The Adiana tubal occlusion method was performed between 2009 and 2012. The mean age of women was 39.3 ± 4.6 years, mean BMI was 25.4 ± 5.2. Of the included women 9.7% were nulligravida and 13% were nullipara. Patient characteristics are presented in Table I. In total 300 women were eligible for Adiana tubal occlusion. In eight women device placement, after the hysteroscopic onset was not attempted. Reasons for this were suspicion of uterine anomaly, intrauterine adhesions in two cases, and no visible tubal ostia in five cases.

**Table I:** Patient characteristics.

	Women undergoing Adiana placement procedure (n=300)
	Mean ± SD
Age [Years]	39.3 ± 4.6
BMI	25.4 ± 5.2
	N (%)
Gravidity	
0	29 (9.7%)
≥1	257 (85.6%)
Missing	14 (4.7%)
Parity	
0	39 (13.0%)
≥1	248 (82.7%)
Missing	13 (4.3%)

### Procedure and successful bilateral placement

In the intention-to-treat group, 255 out of 292 women (87.3%) had successfully placed devices in both fallopian tubes after the first procedure. In 37 women the first attempt failed, and a second procedure was performed in 21 due to the earlier placement failure. This second attempt was successful in 16 cases (76.2%). Two women had a third attempt to achieve a successful placement. The main reasons for a second or third attempt were tubal spasm, non-visible tubal ostia due to oedema or technical problems. Causes for placement failure are listed in Table II.

**Table II:** Reasons for procedure failure.

Reason	N (%)
Intracavitary abnormality removal during first attempt	2 (4.8%)
Technical problem / Catheter could not be advanced/loss of contact	11 (26.2%)
Tubal spasm/blockage	12 (28.6%)
Oedema / ostium not visible	8 (19%)
Adhesions/synechiae/niche	2 (4.8%)
Too lateral ostium (could not be reached)	4 (9.5%)
Unknown	3 (7.1%)
Total	42

**Figure 1:**
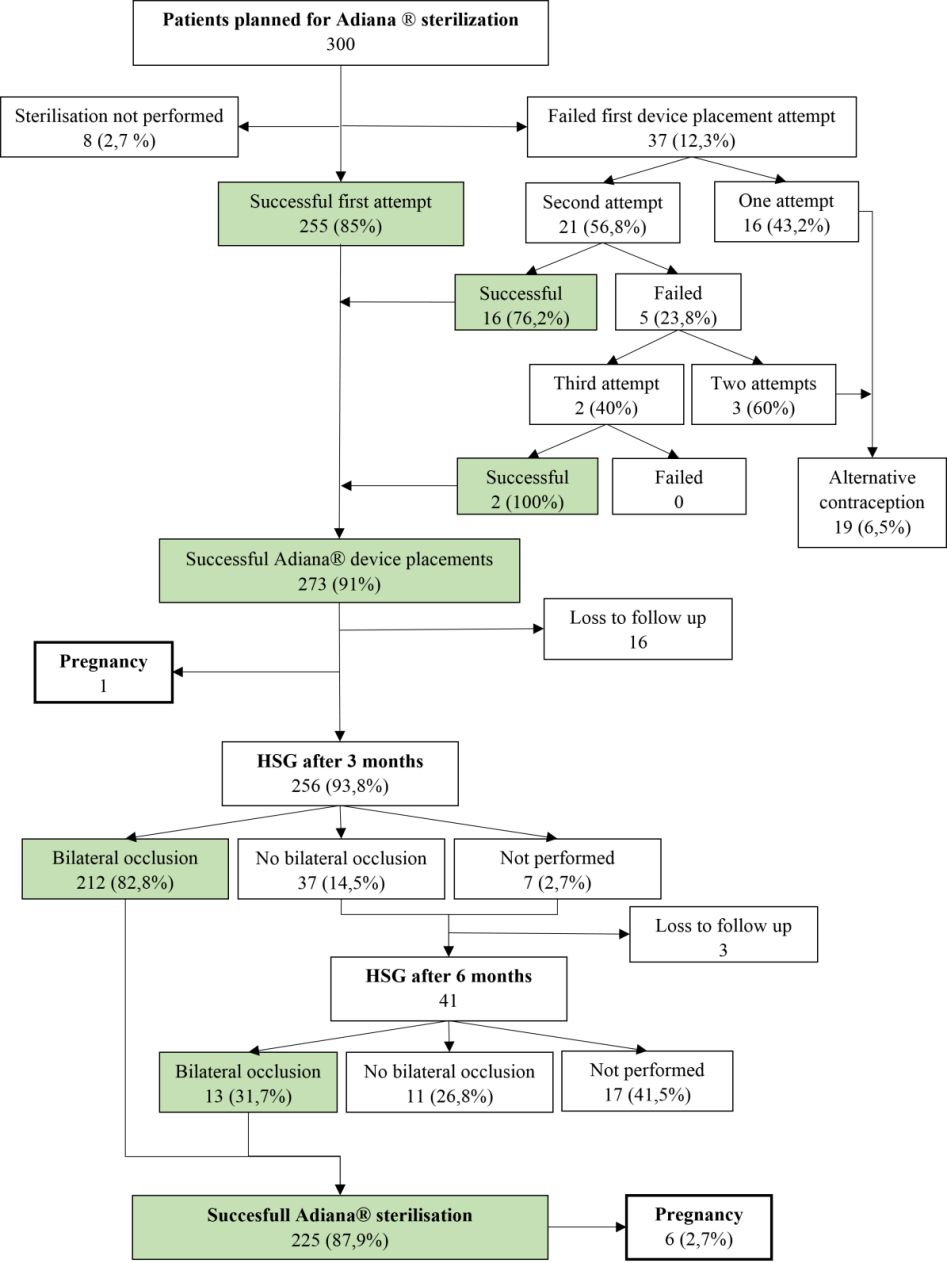
Flowchart presenting Adiana placement and hysterosalpingography (HSG) outcome.

The reasons for no further placement attempts in 16 women who had one unsuccessful placement were not recorded. Three women had two Adiana placement attempts without a successful placement. In total, 273 women out of 292 (93.5%) ultimately had a successful bilateral Adiana device placement (Figure 1).

### Bilateral occlusion confirmed with HSG

Of the 273 women with successful bilateral placement, 16 were lost to follow-up and one woman became pregnant during the three months follow-up. Thus, 256 women (93.8%) were evaluated with an HSG. Bilateral occlusion was confirmed in 212 women (82.8%). These women were instructed to rely on Adiana tubal occlusion. In seven cases (2.7%) HSG was not performed. The reason for this was a failure to attend the appointment in four cases and the three other cases are unknown. A total of 37/256 women (14.5%) did not have bilateral occlusion. An HSG after 6 months was indicated if the first was not performed or deemed inconclusive and if a bilateral occlusion had not yet been established. This was indicated for 44 women, performed in 41 women, and bilateral occlusion was confirmed in 13 additional women. This means 225 out of 256 successful bilateral placements (success rate of 87.9%) according to the HSG outcome. Of the 292 women in the intention-to-treat group, 225 (77.1%) were ultimately able to rely on the Adiana device (Figure 1). The second HSG was not performed in 17 cases, of whom four women did not attend the appointment, seven women used an alternative contraceptive, and six cases are unknown. The group of seven women with an alternative contraceptive consisted of four laparoscopic sterilisations, two sterilisations with Essure and one other form of contraceptive. Eleven women did not have bilateral tubal occlusion after six months, four of them underwent laparoscopic sterilisation, two used an intra-uterine device, and one was lost to follow-up. Of four women the alternative contraception was unknown.

### Complications

Of the 315 hysteroscopic procedures performed in total, complications were reported in 14 women (4.4%). All complications were minor and resolved without additional interventions. Complications or side effects included nausea in five women, vasovagal reaction in five women and one suspicion of perforation. In three cases the complication was not specified. See Table III for the Clavien-Dindo classification.

**Table III:** Complications.

Clavien-Dindo	N (%)
Grade I	11 (3.5%)
Grade II	0
Grade III	0
Grade IV	0
Grade V	0
Unknown	3 (1%)
Total	14

### Pregnancy

In the study period, seven pregnancies were reported. One pregnancy occurred while waiting for the three-month HSG to confirm tubal occlusion. An ultrasound was made in which the presence of the device could not be confirmed. The other six pregnancies occurred after an HSG which showed bilateral occlusion of both fallopian tubes.

From 2018 to 2020 questionnaires (N=300) were sent to all women in the Netherlands with Adiana devices. A total of 100 women responded to the follow-up questionnaires, a response rate of 33.3%. Out of 100 women, three reported an unplanned pregnancy after Adiana sterilisation. One pregnancy was already known from previous data, the other two were not included in the previous follow-up period. Thus, 3.6% of women (8/225) who were told to rely on Adiana tubal occlusion did become pregnant.

## Discussion

We present the results of a prospective multicentre, single-arm clinical study of the Adiana hysteroscopic tubal occlusion device in the Netherlands. The study was conducted from 2009 to 2012 and currently, Adiana is no longer available on the market. It is important to publish these results years later to learn from previous hysteroscopic techniques. Currently, all hysteroscopic sterilisation techniques are no longer available on the market. The Essure system withdrew from the market in Europe in 2017 and in the United States in 2019 due to reduced sales numbers after safety concerns had arisen (FDA, 2022). Ovabloc was withdrawn from the market in 2009 due to the difficulty of the procedure, disappointing results, technical problems with cold storage of silicon and the irreversibility of the sterilisation method based on histological studies (Veersema, 2015; La Chapelle et al., 2015). This latter feature, of course, was not different from other hysteroscopic sterilisation techniques but contrasted with initial claims stated by the Ovabloc company.

The primary outcome of this study was bilateral tubal occlusion confirmed by HSG, three or six months after placement. The success rate of bilateral occlusion in this study was 77.1% (after 6 months) in the intention-to-treat group. In the Adiana evaluation study, the EASE study, a success rate of 88.4% was reported in the intention- to-treat group (Vancaillie et al., 2008; Anderson and Vancaillie, 2011). The lower success rate in this study cannot be fully explained, but it is also low compared to other hysteroscopic devices. With Essure and Ovabloc sterilisation, the probability of confirmed correct placement was 90-100% and 91- 100%, respectively (La Chapelle et al., 2015).

Since there seemed to be a trend towards a difference in success rate between centres depending on the case volume (albeit not significant), perhaps one of the reasons for these conflicting data may have been a subtle difference in experience. The requirement to place the device exactly at the same location as the inflicted coagulation effect could perhaps have made the procedure too challenging for the average physician.

Of the women who could rely on Adiana tubal occlusion, 3.6% became pregnant. The publication by Anderson and Vancaillie (2011) found a pregnancy rate of 1.6%. Compared to the pregnancy rate of Essure of 0.1% and Ovabloc of 1%, the Adiana pregnancy rate of 1.6-3.6% is much higher and probably to be considered unacceptable (La Chapelle et al., 2015).

A sterilisation was concluded to be successful based on the HSG. However, eight women became pregnant after a positive HSG after three months. It is possible that the confirmation test, on which the sterilisation device was not visible, was not specific enough to demonstrate tubal occlusion. The occlusion of the fallopian tubes as shown on HSG can also be due to spasm of the fallopian tubes, which can cause false positive results. HSGs with false positive occlusion are widely reported in the literature. In a study of 40 infertile women with proximal tubal occlusion seen at the first exam, repeat HSG demonstrated patency in 60% of cases (Dessole et al., 2000). In addition, the Adiana device initially was not radiopaque and therefore not visible on the HSG, meaning the presence and location of the devices could not be assessed. Although the devices are visible on ultrasound, this imaging method alone cannot demonstrate patency or occlusion of the fallopian tubes, and the ultrasound was not included in the study protocol. Research has been performed on hysterosalpingo- foam sonography for tubal examination in fertility work-up, but not as a confirmation test for showing occlusion after sterilisation (van Welie et al., 2022). One woman became pregnant before her three months HSG. The ultrasound performed showed no device on one side, which may be due to an expulsion, explaining the unintended pregnancy. A newer version of Adiana was radiopaque, but the device was withdrawn from the market shortly after this version became available. Radiopacity of the device is highly recommended for future research and development of hysteroscopic sterilisation devices (Martin and Evantash, 2011). Ideally, it should demonstrate tubal occlusion immediately, both with HSG and with ultrasound or foam ultrasound.

Starting from 2014, safety concerns about Essure sterilisation devices were raised. Women reported multiple symptoms potentially attributed to the device. Symptoms varied from abdominal or pelvic pain, heavy menstrual bleeding, psychological complaints and more. These symptoms resulted in large numbers of patients requesting surgical removal of the devices. Until now, the symptoms cannot be fully explained. Bayer stopped selling Essure by July 2017 in Europe and by January 2019 in the United States. Evidence about long- term follow-up of patients with Essure devices is scarce. The phase III study had a follow-up time of 5 years and only three severe adverse events were reported in 2 subjects possibly related to the inserts (Chudnoff et al., 2015). With new devices pre- clinical and post-clinical trials should evaluate all adverse events. Better quality assessment should prevent us from similar situations as Adiana, Essure and Ovabloc.

A limitation of the study was the length of follow-up time. We tried to overcome this by sending questionnaires years later, however the response rate was only 33.3%. Of these 100 women responding, two new pregnancies occurred. Making it unclear whether there are even more unplanned pregnancies or whether there is a selection bias.

Another limitation is that the study was conducted 10 years ago and therefore information may have been lost. However, it is important to learn from previous techniques on the way to developing a new hysteroscopic sterilisation device. Currently, sterilisation methods with a hysteroscopic route are lacking, which is a loss for women, considering all its benefits. The hysteroscopic route avoids the transabdominal route with a longer recovery time and the risks of complications. The advantage of the reduced need for anaesthesia, which facilitates sterilisation in an ambulatory setting, is a requirement for a new technique. These advantages make hysteroscopic sterilisation a good option for all women who just wish to avoid incisional surgery or general anaesthesia, especially with contraindications for laparoscopy (La Chapelle et al., 2015). Another significant added benefit is the cost savings realised from both the office setting and the hysteroscopic approach, which amounts to a difference of approximately $2,000 (Connor, 2009). In the development of new hysteroscopic sterilisation devices, the experiences with Adiana should also be considered, as the radiopacity and the possibility of ultrasound control seem to be relevant factors that influence the reliability of the confirmation test.

## Conclusion

This study showed a pregnancy rate of 3.6% with the Adiana permanent contraception system, a rate generally higher than for other hysteroscopic sterilisation techniques. Women no longer have the option of a choice for hysteroscopic sterilisation. When developing a new device, it probably should be radiopaque with the possibility of ultrasound control. This seems to be relevant to the reliability of the confirmation test. In addition, we should strive to enhance feasibility and better pre- and post-clinical studies.
